# Aberrant Right Subclavian Artery Identified before Carotid Artery Stenting

**DOI:** 10.31662/jmaj.2023-0049

**Published:** 2023-06-12

**Authors:** Tatsuya Tanaka, Ryohei Sashida, Yu Hirokawa, Akira Matsuno

**Affiliations:** 1 Department of Neurosurgery, International University of Health and Welfare, School of Medicine, Narita, Japan

**Keywords:** aberrant right subclavian artery, aortic arch anomaly, carotid artery stenting, retroesophageal right subclavian artery, three-dimensional computed tomography angiography

An 84-year-old man with right common iliac artery dissection presented with a complaint of transient right upper limb weakness. Magnetic resonance imaging of the head revealed a cerebral infarct in the left hemisphere, and magnetic resonance angiography revealed severe stenosis of the left internal carotid artery (Figure 1). Antithrombotic therapy was performed, and a transradial approach was selected as the patient had previously undergone iliac artery dissection. Preoperative three-dimensional computed tomography angiography revealed an aberrant right subclavian artery with a retroesophageal course originating from the most distal branch of the aortic arch (Figure 2, arrow). Carotid artery stenting was performed via the left femoral artery.

**Figure 1.**
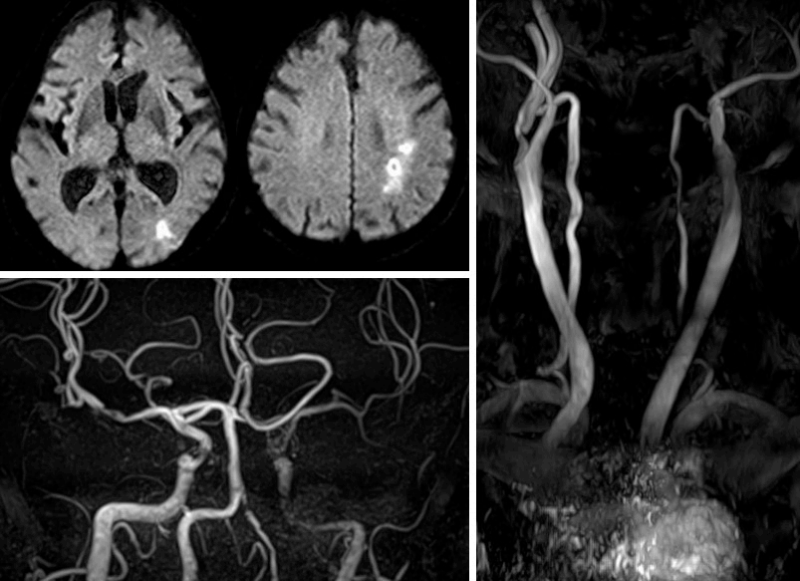
Magnetic resonance imaging of the head showing a cerebral infarct in the left hemisphere and magnetic resonance angiography showing severe stenosis of the left internal carotid artery.

**Figure 2.**
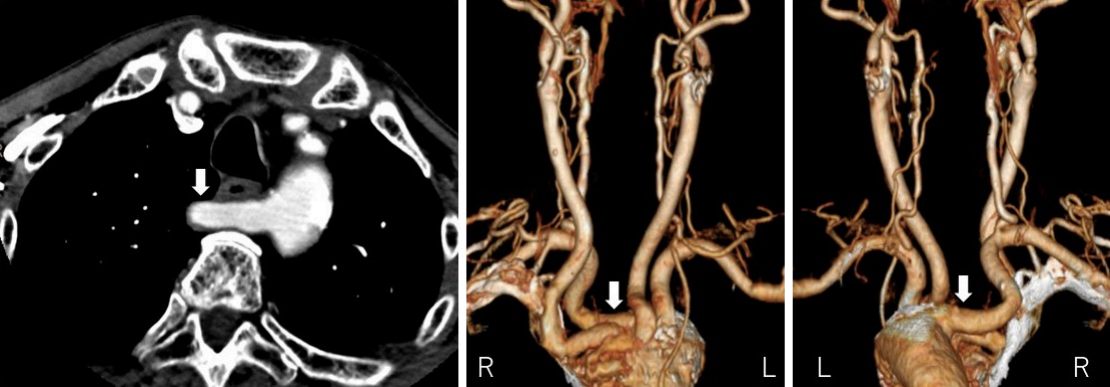
Preoperative three-dimensional computed tomography angiography showing an aberrant right subclavian artery with a retroesophageal course originating from the most distal branch of the aortic arch (arrow).

Aberrant right subclavian artery is a rare aortic arch anomaly, affecting only 0.4%-2.0% of the population ^(1)^. Most patients remain asymptomatic; however, aneurysmal dilatation, aortic dissection, and tracheal and esophageal compression are some serious vascular complications.

Recently, endovascular treatment via the radial or brachial artery has gained more popularity. Preoperative three-dimensional computed tomography angiography alerts physicians about any morphological abnormalities and reduces surgical risks.

## Article Information

### Conflicts of Interest

None

### Author Contributions

TT wrote the first draft and managed the submission process. RS, YH, and AM commented on previous versions of the manuscript. All authors read and approved the final version of the manuscript.

### Informed Consent

We have obtained informed consent for this case report.
